# Methotrexate Chemotherapy Causes Growth Impairments, Vitamin D Deficiency, Bone Loss, and Altered Intestinal Metabolism—Effects of Calcitriol Supplementation

**DOI:** 10.3390/cancers15174367

**Published:** 2023-09-01

**Authors:** Yu-Wen Su, Alice M. C. Lee, Xukang Xu, Belinda Hua, Heather Tapp, Xue-Sen Wen, Cory J. Xian

**Affiliations:** 1 UniSA Clinical and Health Sciences, University of South Australia, Adelaide, SA 5001, Australia; yu-wen.su@unisa.edu.au (Y.-W.S.); alice.lee@unisa.edu.au (A.M.C.L.); xukang.xu@sa.gov.au (X.X.); belinda.hua@sa.gov.au (B.H.); 2 Department of Haematology & Oncology, Women’s and Children’s Hospital, North Adelaide, SA 5006, Australia; heather.tapp@sa.gov.au; 3 School of Pharmaceutical Sciences, Cheeloo College of Medicine, Shandong University, Jinan 250012, China; wsx@sdu.edu.cn

**Keywords:** childhood cancer chemotherapy, vitamin D deficiency, growth impairments, bone loss, vitamin D metabolism

## Abstract

**Simple Summary:**

Vitamin D deficiency or insufficiency is prevalent in childhood cancer patients and survivors; however, the underlying etiology and effectiveness of vitamin D supplementation in preventing chemotherapy-induced bone loss are unclear. This study aimed to use a rat model to investigate whether methotrexate chemotherapy-induced vitamin D deficiency is related to intestinal damage and whether vitamin D supplementation can attenuate the resultant bone loss. We found that methotrexate chemotherapy causes vitamin D deficiency, bone growth impairments, bone loss, and altered intestinal vitamin D metabolism, which are associated with intestinal damage, and vitamin D supplementation inhibits methotrexate-induced bone loss due to its effect in suppressing methotrexate-induced bone lysis. This study has provided a mechanistic insight into vitamin D deficiency caused by methotrexate chemotherapy (which is commonly used in childhood oncology), and it has produced experimental evidence for the effectiveness of vitamin D supplementation in preventing chemotherapy-induced bone loss (commonly found in cancer patients and survivors).

**Abstract:**

Vitamin D deficiency or insufficiency is prevalent in childhood cancer patients and survivors after chemotherapy; further studies are needed to investigate the underlying aetiology and effectiveness of vitamin D supplementation in preventing chemotherapy-induced bone loss. This study used a rat model of treatment with antimetabolite methotrexate to investigate whether methotrexate chemotherapy causes vitamin D deficiency and if vitamin D supplementation attenuates the resultant bone loss. Methotrexate treatment (five daily injections) decreased serum vitamin D levels (from 52 to <30 ng/mL), reduced body and bone lengthening and tibial trabecular bone volume, and altered intestinal vitamin D metabolism, which was associated with intestinal mucosal damage known to cause malabsorption of nutrients, including dietary vitamin D and calcium. During the early stage after chemotherapy, mRNA expression increased for vitamin D activation enzyme CYP27B1 and for calcium-binding protein TRPV6 in the intestine. During the intestinal healing stage, expression of vitamin D catabolism enzyme CYP24 increased, and that of TRPV6 was normalised. Furthermore, subcutaneous calcitriol supplementation diminished methotrexate-induced bone loss due to its effect suppressing methotrexate-induced increased bone resorption. Thus, in young rats, methotrexate chemotherapy causes vitamin D deficiency, growth impairments, bone loss, and altered intestinal vitamin D metabolism, which are associated with intestinal damage, and vitamin D supplementation inhibits methotrexate-induced bone loss.

## 1. Introduction

Chemotherapy as a cancer treatment modality has been shown to exert severe side effects on the skeletal system, which can negatively impact the quality of life of cancer patients and survivors. Previous clinical and basic studies have demonstrated that paediatric cancer chemotherapy, particularly with high-dose glucocorticoids and anti-metabolite methotrexate (MTX), is a major risk factor for cancer treatment-induced bone defects (including growth impairments, bone loss, and fractures) in patients or survivors of childhood cancers, particularly acute lymphoblastic leukaemia (ALL) [1,2,3,4,5]. Altered bone metabolism, including reduced bone formation, increased bone resorption, and reductions in bone mass or bone mineral density (BMD), has been reported in ALL patients during/after chemotherapy and even in long-term survivors of ALL [6,7,8]. Previous studies have suggested that chemotherapy-induced bone defects are likely to be attributed to multiple factors, such as impaired nutrient absorption and malnutrition resulting from chemotherapy-induced intestinal mucosa damage (mucositis) as well as altered bone modelling/remodelling processes (reduced bone formation and increased bone resorption) resulting from the direct or indirect actions of chemotherapy on bone formation cells (osteoblasts), bone resorption cells (osteoclasts) and bone maintenance cells (osteocytes) [3,5,9].

Vitamin D, a steroid hormone, is well known to play a critical role in regulating calcium (Ca) homeostasis, bone development, and bone metabolism, and an adequate serum level of 25-hydroxyvitamin D [25(OH)D] has been shown to help to protect against osteoporosis and to reduce risks of fractures [10]. Due to this critical role of vitamin D in regulating Ca homeostasis and bone metabolism, there have been many clinical studies on vitamin D status in paediatric cancer patients and survivors and on the potential effects of vitamin D supplementation. Compared to the issue of vitamin D inadequacy or deficiency in healthy children/adolescents, vitamin D deficiency/inadequacy has been suggested to be at an even higher risk in paediatric cancer patients [11,12,13]. While lower vitamin D levels and bone formation were reported in patients newly diagnosed with ALL [9], a meta-analysis showed that the prevalence of serum 25(OH)D deficiency or insufficiency was higher during or at the end of cancer treatment compared to the time of ALL diagnosis [13]. Lower plasma levels of 1,25-dihydroxyvitamin D [1,25(OH)2D] were found in >70% of ALL patients during 24 months of chemotherapy [14]. Vitamin D deficiency has been found in most of ALL patients at the end of glucocorticoid induction treatment (85%) [15,16]. Vitamin D deficiency was also found in ALL patients receiving high-dose methotrexate chemotherapy [17]. Due to the prevalence of vitamin D deficiency/insufficiency and lower BMD in ALL patients and survivors, vitamin D and calcium supplementation during ALL treatment have been considered, recommended, or given to childhood cancer patients and survivors in some clinical studies [7,18,19,20,21]. 

However, although there have been many clinical studies demonstrating lower plasma levels of vitamin D in ALL patients and survivors as described above, the underlying aetiology is unclear, which needs further mechanistic studies with animal models. Furthermore, while previous clinical studies have not been able to show clear evidence for the effectiveness of vitamin D supplementation in improving the bone density of ALL survivors (probably due to a paucity of cases/studies and the heterogeneity of the therapeutic regimens) [20,21], animal model studies are needed to demonstrate potential effects of vitamin D supplementation in attenuating bone loss caused by chemotherapy. Since increasing the intestinal absorption of ingested calcium is central to the maintenance of calcium homeostasis by vitamin D [22], the current study used a rat model of MTX treatment (used commonly in childhood oncology) to demonstrate whether MTX chemotherapy can cause vitamin D deficiency, changes in body/bone lengthening and bone turnover, and alterations in intestinal tissue vitamin D metabolism, and if vitamin D supplementation can attenuate MTX-induced bone loss.

## 2. Materials and Methods

### 2.1. Animal Studies

#### 2.1.1. MTX Treatment Time Course Study

**Figure 1:**
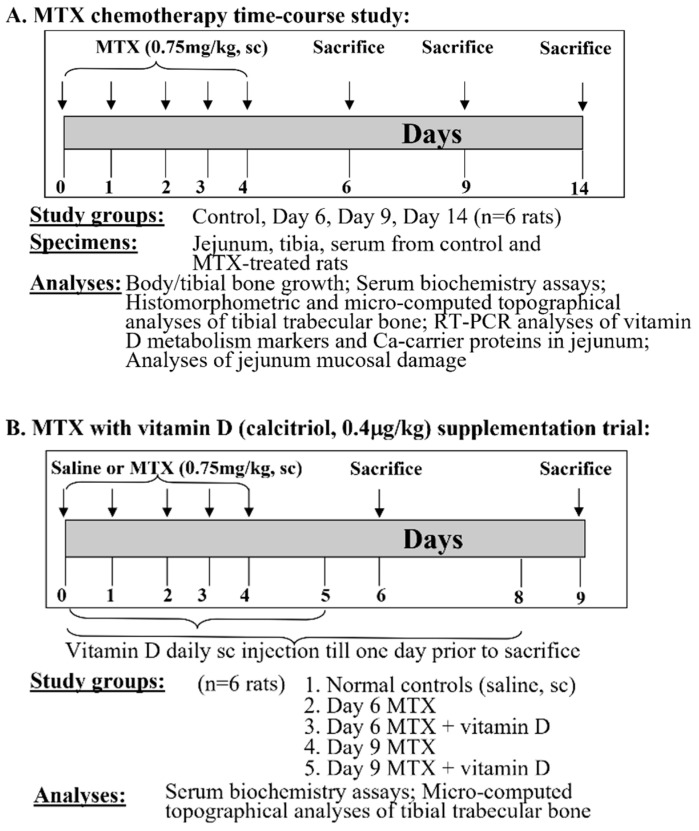
Design of this study. (**A**) Methotrexate (MTX) chemotherapy time-course study and (**B**) vitamin D (calcitriol) supplementation trial. Both MTX and vitamin D were given daily subcutaneously (sc) at doses specified.

Sprague Dawley male rats (6 weeks old, with body weights of around 145 g) were housed at 24 °C with a 12-h light/dark cycle. They were supplied ad libitum with tap water and a commercial standard rat chow diet containing 0.8% calcium, 0.7% phosphorus, and 2000 U/kg vitamin D (cholecalciferol) (Specialty Feeds, Glen Forrest, WA, Australia). MTX (M9929, Sigma-Aldrich, St. Louis, MO, USA) was dissolved firstly in dimethyl sulfoxide (Sigma-Aldrich) and then diluted in saline before use. Rats were weighed daily and received MTX at 0.75 mg/kg subcutaneously (sc) once daily for 5 consecutive days (mimicking the intense MTX treatment for ALL) as described in [23,24]. Groups of MTX-treated rats were euthanised by CO_2_ overdose on days 6, 9, and 14 after the initial MTX dose (n = 6 rats/group), the time points which were previously found to have partial (day 6) and significant (day 9) histological damages and near normal recovery (day 14) in tibial metaphysis bone of treated rats [23]. A group of saline-injected rats was also euthanised on the same day as the MTX-treated day 14 rats, which served as the control group for growth measurements alongside the MTX day 14 group. For the control rats and MTX-treated rats euthanised on day 14, the total body lengths were measured (from the tip of the nose to the tip of the tail) at the time of the first MTX injection and at euthanasia, and the lengths of the left tibia were also measured at sample collection. Serum, specimens of the proximal region of the small intestine (jejunum), and tibial bones were collected for biochemical, structural, and gene expression analyses [23,25] (Figure 1A).

#### 2.1.2. MTX Treatment and Vitamin D Supplementation Trial

For the vitamin D supplementation trial, MTX treatment was given as described above, and calcitriol (the active form of vitamin D) (D1530, Sigma-Aldrich, St. Louis, MO, USA) (firstly dissolved in 90% ethanol and then diluted in saline before use) was administered subcutaneously once daily at 0.4 μg/kg body weight [26] both during and after the MTX treatment period until one day before being euthanised. Groups of rats were euthanised on days 6 and 9 after the initial MTX injection (n = 6 rats/group). A group of saline-injected rats was also euthanised on the same day as the MTX-treated day 9 rats, which served as normal controls. Serum and tibial bone specimens were collected [23], respectively, for biochemical and structural analyses (Figure 1B). The above protocols followed the Australian Code of Practice for the Care and Use of Animals and were approved by the Animal Ethics Committee of the University of South Australia (approval number: U4-14).

### 2.2. Serum Biochemistry Assays

Serum 25(OH)D levels (ng/mL) were measured using a rat-specific 25(OH)D enzyme-linked immunosorbent assay (ELISA) kit as instructed (MBS728692, MyBioSource, San Diego, CA, USA). Serum levels of calcium (Ca, mmol/L) and alkaline phosphatase (ALP, U/L, commonly used as a bone formation marker) were measured with an automated KoneLab Clinical Chemistry Analyser using generic reagents (981359, Thermo Fisher Scientific, Thebarton, SA, Australia) [27,28]. Serum levels of bone resorption marker collagen type I cross-linked C-telopeptide (CTX-1) were measured using a rat-specific CTX-1 ELISA kit (CSB-E12776r, Cusabio, Hubei, China) [29].

### 2.3. Histomorphometric and Micro-Computed Topographical (μ-CT) Analyses of Tibial Trabecular Bone 

For histological studies, the left proximal tibiae of control rats or MTX-treated rats were fixed in 10% formalin for 24 h, decalcified in Immunocal (Decal Corp, Tallman, NY, USA) at 4 °C, processed and embedded in paraffin wax. On hematoxylin- and eosin- (H&E)-stained sections, trabecular number, thickness, spacing, and bone volume fraction (trabecular bone volume/total tissue volume, BV/TV%) were analysed as described [23]. In addition, left proximal tibial bone samples from the vitamin D supplementary trial were fixed in formalin and stored in 70% ethanol at 4 °C prior to being analysed using a Skyscan 1172 μ-CT system (Skyscan, Antwerp, Belgium) at an 11.2 μm/pixel resolution to calculate trabecular bone volume fraction, trabecular thickness, and spacing [30]. Analyses were performed at a 2 mm region of interest in the metaphysis trabecular network starting from 2 mm below the growth plate [30]. 

### 2.4. RT-PCR Analyses of Vitamin D Metabolism Markers and Ca-Carrier Proteins in Jejunum

Isolation of total RNA from frozen jejunum specimens was performed using a phenol/chloroform extraction method using TRIzol reagent as instructed (Invitrogen, Thermo Fisher Scientific, Thebarton, Australia). Following a DNase digestion step, the RNA was used to synthesise the first strand of cDNA using random decamers (GeneWorks, Adelaide, Australia) and Super-Script™ III RT kit (Invitrogen). SYBR green-based PCR assays were then performed using specific primer pairs for target genes and endogenous control gene Cyclophilin A (supplied by GeneWorks, Adelaide, Australia) [31]. Vitamin D metabolism-related molecules analysed for mRNA expression included TRPV6 (transient receptor potential cation channel Vanilloid subfamily member 6), CALBINDIND9k (calcium-binding protein D9k), CYP27B1 (cytochrome P450 family 27 subfamily B member 1), and CYP24 (P450 family 24 subfamily A member 1). Primer sequences were described previously for CYP27B1 and CYP24 [32] and for TRPV6 and CALBINDIND9k [33]. mRNA expression levels of these target genes were expressed as ratios to the levels of Cyclophilin A. 

### 2.5. Analyses of MTX-Induced Small Intestine Mucosal Damage and Recovery 

For assessing intestinal mucosal damage and repair over the time course after MTX treatment, changes in villus height and crypt depth were measured by image analyses on hematoxylin-stained transverse paraffin-embedded tissue sections (4 µm in thickness) of the proximal jejunum. Microscopic images were acquired and analysed as described [34,35]. As an additional means to assess intestinal damage and repair, numbers of goblet cells (cells that secrete mucin for protecting intestinal mucosa and being a marker for intestinal functional recovery) in the intestinal villi were also assessed on 4-μm paraffin sections of the proximal jejunum. Sections were first stained by the periodic acid-Schiff (PAS) technique that stains mucin, and PAS-stained goblet cells in the villi were then counted and expressed as goblet cells/mm villous length as described [35]. Furthermore, sucrase activities (μmol glucose/well/min/g of tissue) in intestinal samples were assessed as described [34,36]. Briefly, jejunum segments were homogenised in 10 mM PBS, and supernatants from the homogenates (after being centrifuged at 3500 rpm at 4 °C for 10 min) were diluted 1:50 in 50 mM PBS prior to being used for the sucrase activity assays with a standard curve prepared using glucose standards in 96-well plates.

### 2.6. Statistical Analyses

Statistical analyses of data (presented as mean +/-SEM) were performed using SPSS Version 25 (IBM, New York, NY, USA) and GraphPad Prism Version 8.1.2 (GraphPad, La Jolla, CA, USA). ANOVA was carried out, followed by Bonferroni’s or Tukey’s post hoc test for multiple comparisons. Specifically, one-way ANOVA was used to analyse data on the body weight changes, body length gain, and tibial length, comparing the control rats and the rats treated with MTX and sacrificed on day 14. Two-way ANOVA was applied for data from time-course studies without vitamin D intervention, as well as for data from the vitamin D intervention study but without different time points. Three-way ANOVA was used for data with different time points and vitamin D intervention. Every possible or relevant comparison between the study groups was considered. A *p*-value of <0.05 was considered statistically significant. 

## 3. Results

### 3.1. MTX Chemotherapy Caused Body Weight Loss and Decreased Total Body Length Gain and Tibial Length

**Figure 2:**
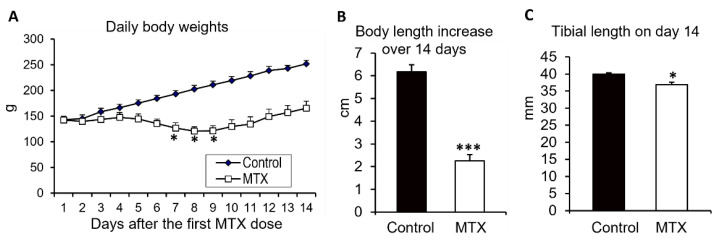
Effects of methotrexate (MTX) chemotherapy (5 once-daily injections at 0.75 mg/kg) on (**A**) daily body weights, (**B**) total body length gain over the 14-day experimental period, and (**C**) the total tibial lengths of control rats or MTX-treated rats euthanised on day 14 after the first MTX dosing. One-way ANOVA was used for data analyses (n = 6). * *p* < 0.05 and *** *p* < 0.001 compared to the initial body weights at the start of MTX dosing or to the control group on day 14.

Firstly, the effects of MTX chemotherapy were examined on daily body weight changes, total body length gain over the 14-day experimental period, and the final tibial length on day 14 (after the first MTX dose) for the control rats and the MTX-treated rats that were euthanised on day 14 (Figure 2). As it occurs in children who receive intensive MTX dosing for cancer treatment, MTX-treated rats in the current study showed a reduced appetite during the MTX treatment period, and the rats had significant body weight losses during days 7 and 9 after the first MTX dose (*p* < 0.05 compared to the original weight on day 0), with day 8 recording the worst total loss of 15% compared to the original weight. The rats started to gain weight again on day 10 and recovered to their original body weights by day 12 (Figure 2A).

Consistent with the adverse effects on body weights, MTX-treated rats also had a significantly lower gain in the total body length (from the tip of the nose to the tip of the tail) over the 14-day experimental period compared to the control rats (2.3 cm vs. 6.2 cm, *p* < 0.001) (Figure 2B). In addition, on day 14, the tibiae of MTX-treated rats were shorter (36.9 mm vs. 40 mm, *p* < 0.05) (Figure 2C). 

### 3.2. MTX Chemotherapy Reduced Serum Vitamin D Levels, Altered Bone Turnover, and Decreased Tibial Trabecular Bone Volume

**Figure 3:**
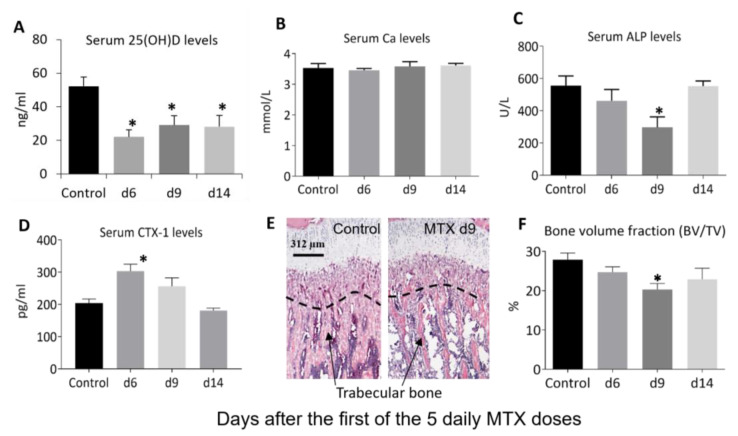
Effects of methotrexate (MTX) chemotherapy (5 once-daily injections at 0.75 mg/kg) on serum levels of (**A**) vitamin D (25(OH)D), (**B**) calcium (Ca), and markers of bone turnover, including (**C**) alkaline phosphatase (ALP) and (**D**) collagen type I cross-linked C-telopeptide or CTX-1), as well as proximal tibial metaphysis bone (**E**) morphology as shown on microscopic images of hematoxylin- and eosin-stained sections (showing loss of trabecular bone particularly within the secondary spongiosa—the region below the dashed line, with arrows pointing to bone trabeculae; scale bar = 312 μm), and (**F**) bone volume fraction (bone volume/total tissue volume, BV/TV), on days (d) 6, 9, and 14 after the first MTX dosing. Two-way ANOVA was used for data analyses (n = 6). * *p* < 0.05 compared to the saline-treated control group.

Next, we examined the effects of MTX chemotherapy on levels of serum vitamin D and markers of bone turnover as well as bone volume (Figure 3). On days 6, 9, and 14 after the first MTX dose, serum vitamin D (25(OH)D) levels (22–29 ng/mL) were significantly lower compared to the normal level (52 ng/mL) (*p* < 0.05 for all time points), being 42%, 58%, and 61% of the normal control, respectively (Figure 3A). This can be classified as a deficiency as they are lower than the human “deficient level” of <30 ng/mL accepted by many authors [37,38].

Despite the reduced serum levels of vitamin D in rats after chemotherapy, the serum Ca levels showed no obvious changes at all the time points examined after MTX treatment (*p* > 0.05 vs. control) (Figure 3B). On the other hand, serum markers of bone turnover displayed significant changes after MTX chemotherapy. Serum levels of the bone formation marker ALP showed a tendency to reduce on day 6 (*p* > 0.05 vs. control), were significantly lower compared to the normal level on day 9 (being 53% of the control, *p* < 0.05) and returned to normal on day 14 (*p* > 0.05 vs. control) (Figure 3C). Levels of the bone resorption marker CTX-1 were significantly higher compared to the normal level on day 6 (being 149% of the control, *p* < 0.05), showed a tendency to increase on day 9 (being 126% of the control level, *p* > 0.05), and returned to the normal level on day 14 (*p* > 0.05 vs. control) (Figure 3D). Consistent with the obvious changes in serum levels of bone turnover markers, local histomorphometry analyses (Figure 3E) of the proximal tibia showed a tendency to reduce trabecular bone volume on day 6 (being 88% of the control, *p* > 0.05), a significant decrease on day 9 (being 73% of the control, *p* < 0.05), and a partial recovery on day 14 (82% of the control, *p* > 0.05) (Figure 3F). 

### 3.3. MTX Chemotherapy Altered Intestinal Expression of Key Vitamin D Metabolism Enzymes and Ca-Carrier Proteins, which Was Associated with MTX-Induced Intestinal Mucosa Damage

**Figure 4:**
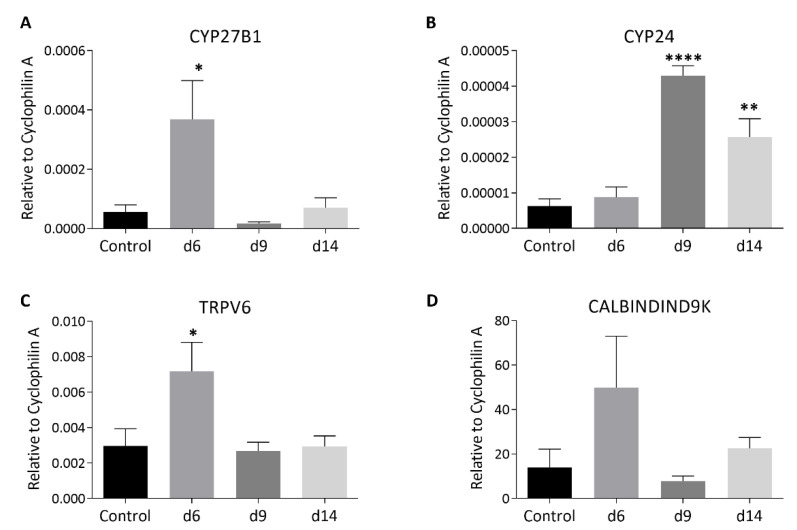
Effects of methotrexate (MTX) chemotherapy (5 once-daily injections at 0.75 mg/kg) on mRNA expression levels of vitamin D metabolism or calcium absorption-related molecules, including (**A**) CYP27B1 (cytochrome P450 family 27 subfamily B member 1), (**B**) CYP24 (P450 family 24 subfamily A member 1), (**C**) TRPV6 (transient receptor potential cation channel Vanilloid subfamily member 6), and (**D**) CALBINDIND9k (calcium-binding protein D9k), in the jejunum samples collected on days (d) 6, 9, and 14 after the first MTX dosing. Two-way ANOVA was used for data analyses (n = 6). * *p* < 0.05, ** *p* < 0.01, and **** *p* < 0.0001 compared to the saline-treated control group.

In the normal state, increasing the intestinal absorption of ingested calcium is central to the maintenance of calcium homeostasis by vitamin D [22]. This is via increasing local synthesis of calcium-carrying proteins, including TRPV6 and CALBINDIND9K [39,40]. In addition, vitamin D metabolism has been found to occur locally in the intestine [41,42]. Thus, the current study has examined the effect of MTX chemotherapy on intestinal expression of the key calcium-carrying proteins TRPV6 and CALBINDIND9K as well as the key metabolism enzymes CYP27B1 (responsible for converting the inactive 25(OH)D to the active hormonal form of 1,25(OH)2D) and CYP24 or CYP24A1 (the major vitamin D 24-hydroxylase that catabolises 25(OH)D and 1,25(OH)2D) [42,43] (Figure 4). On day 6 after MTX chemotherapy, there were significant increases in mRNA expression of the key activating enzyme CYP27B1 (*p* < 0.05 vs. control) (Figure 4A) and of the key Ca-carrying protein TRPV6 (*p* < 0.05 vs. control) (Figure 4C) (as well as a trend of an increase in CALBINDIND9K, *p* > 0.05 vs. control) (Figure 4D). Of note, the high level of CALBINDIND9K mRNA expression in the canine proximal small intestine has also been found previously [44]. However, the increases in expression of these molecules returned to normal levels on days 9 and 14 (*p* > 0.05 vs. control). On the other hand, interestingly, levels of expression of the vitamin D catabolising enzyme CYP24 were found to have no changes on day 6 (*p* > 0.05 vs. control) but increased significantly on days 9 (*p* < 0.0001 vs. control) and 14 (*p* < 0.01 vs. control) (Figure 4B). These changes suggest an increase in activating 25(OH)D to 1,25(OH)2D and in Ca absorption demand locally in the intestine during the early stage after MTX chemotherapy, which returns to normal levels during the later recovery stage.

**Figure 5:**
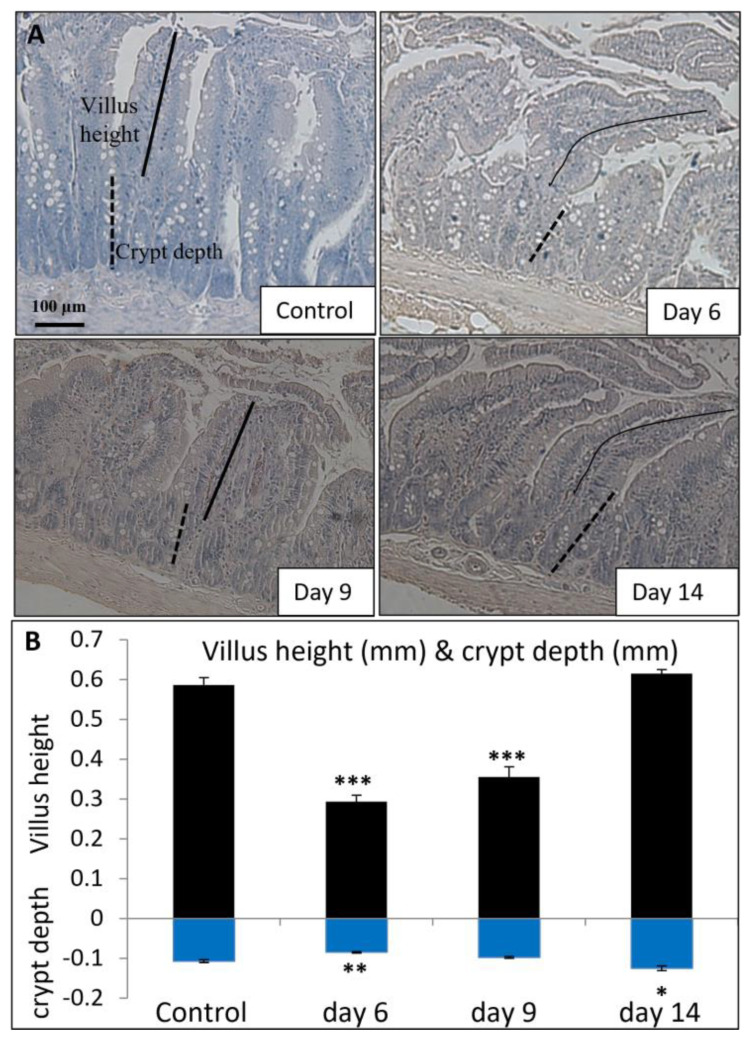
Effects of methotrexate (MTX) chemotherapy (5 once-daily injections at 0.75 mg/kg) on (**A**) histological structure of proximal jejunum (as shown by microscopic images of hematoxylin-stained transverse sections; scale bar = 100 μm) and (**B**) the quantitative histological measurements of villus height (indicated by solid lines) and crypt depth (indicated by dashed lines), in control rats or MTX-treated rats on days 6, 9, and 14 after the first MTX dosing. Two-way ANOVA was used for data analyses (n = 6). * *p* < 0.05, ** *p* < 0.01, and *** *p* < 0.001 compared to the control group.

To examine whether the changes in vitamin D metabolism enzymes and Ca-binding proteins were associated with MTX-induced intestinal damage and subsequent repair, the current study further examined MTX chemotherapy-induced changes in mucosal structure in the small intestine (jejunum segment) on days 6, 9, and 14 after the first of the five daily MTX injections (Figure 5A). A significant shortening of jejunal mucosal villi was observed on day 6 (*p* < 0.001 vs. control), which showed a partial recovery on day 9 (despite still being *p* < 0.001 vs. control) and a complete recovery on day 14 (*p* > 0.05 vs. control) (Figure 5B). There was also a significant reduction in the crypt depth on day 6, which returned to normal on day 9 (*p* > 0.05 vs. control) and appeared overshot on day 14 (*p* < 0.05 vs. control) (Figure 5B).

**Figure 6:**
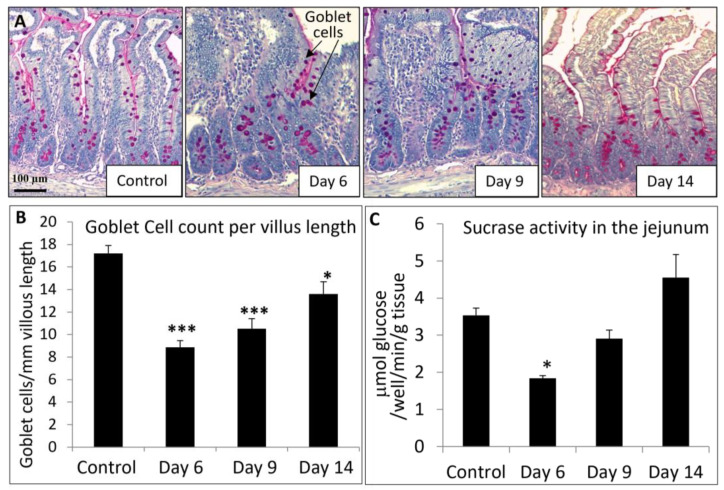
Effects of methotrexate (MTX) chemotherapy (5 once-daily injections at 0.75 mg/kg) on (**A**) goblet cell population in proximal jejunum (as shown by cells with pink colour periodic acid-Schiff mucin stain on the microscopic images, indicated by arrows; scale bar = 100 μm), (**B**) quantitative histological goblet cell counts in the jejunum villi, and (**C**) sucrase activity in jejunum, in control rats or MTX-treated rats on days 6, 9, and 14 after the first MTX dosing. Two-way ANOVA was used for data analyses (n = 6). * *p* < 0.05 and *** *p* < 0.001 compared to the control group.

Consistent with the mucosal structural changes described above, MTX chemotherapy also resulted in a time course of changes in some indicators of intestine damage/repair and function (presence of goblet cells and sucrase activity) in the jejunum. As shown by PAS staining (Figure 6A) and quantification of stained cells, there was a significant loss of goblet cells in the jejunal villi on days 6 (51% of control, *p* < 0.001) and 9 (61% of control, *p* < 0.001) but a significant recovery on day 14 (79% of control, *p* < 0.05) (Figure 6B). Furthermore, as a measure of changes in intestinal digestive function, MTX treatment caused a significant decrease on day 6 (52% of control, *p* < 0.05) in the activity of sucrase (an intestinal brush-border hydrolase that catalyses the hydrolysis of sucrose), which returned towards the normal level on days 9 (82% of control, *p* > 0.05) and 14 (128% of control, *p* > 0.05) (Figure 6C). These results suggest that acute MTX chemotherapy causes significant transient changes in mucosal structure, goblet cell population, and hydrolase sucrase activity in the intestinal mucosa.

### 3.4. Vitamin D Supplementation Inhibited MTX Chemotherapy-Induced Bone Loss due to Its Effect in Suppressing Bone Resorption

**Figure 7:**
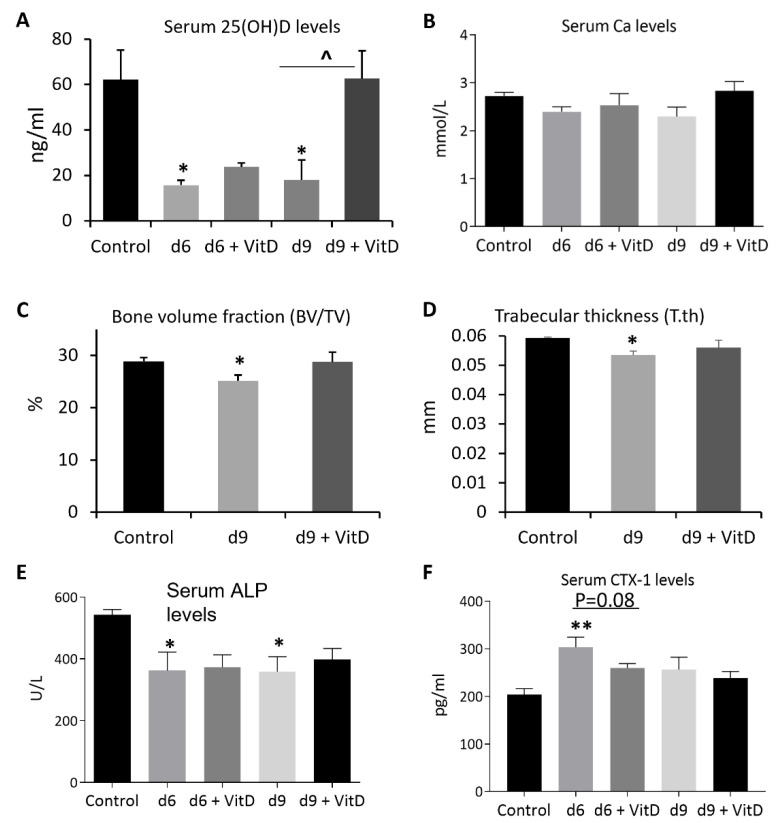
Effects of methotrexate (MTX) chemotherapy (5 once-daily injections at 0.75 mg/kg) with or without supplementation of active form of vitamin D (+ VitD) (calcitriol given subcutaneously once daily at 0.4 mg/kg until one day prior to being sacrificed) on levels of (**A**) serum vitamin D (25(OH)D), (**B**) serum calcium (Ca), (**C**) bone volume fraction (bone volume/total tissue volume, BV/TV), and (**D**) trabecular bone thickness (T.th) of proximal left tibia, as well as levels of serum markers of bone turnover: (**E**) alkaline phosphatase or ALP and (**F**) collagen type I cross-linked C-telopeptide or CTX-1), on days (d) 6, 9, and/or 14 after the first MTX dosing. Two-way ANOVA was used to analyse data without different time points, and three-way ANOVA was used to analyse data with different time points (n = 6). * *p* < 0.05 or ** *p* < 0.01 compared to the control group, and ^ *p* < 0.05 or *p* = 0.08 comparing the two groups under a horizontal bar.

Considering the significant reduction in serum vitamin D levels and obvious trabecular bone loss after MTX chemotherapy as described above, we next investigated whether supplementation of vitamin D could attenuate MTX treatment-induced vitamin D deficiency and bone loss. After calcitriol, the active form of vitamin D, was administered subcutaneously once daily at 0.4 mg/kg [26] during the MTX treatment period and until one day before being sacrificed, on day 6, serum vitamin D levels were found to be only partially restored after supplementation (38% of control) compared to the no supplementation group (25% of control) (*p* > 0.05). However, on day 9, serum vitamin D levels were found to be fully restored after supplementation (100% of control), which were significantly higher compared to the no supplementation group (29% of control) (*p* < 0.05) (Figure 7A). When serum Ca levels were examined, vitamin D supplementation was found to have a marginal effect in increasing Ca levels on day 9 (104% of control) when compared to the no supplementation group (85% of control) (*p* > 0.05) and to have no obvious effects on day 6 (93% vs. 89% of control, respectively) (Figure 7B).

When treatment effects on proximal tibial local trabecular bone volume fractions (BV/TV%) were examined by μ-CT analyses, a significant decrease in BV/TV% was found on day 9 when no vitamin D was supplemented (being 87% of the control) (*p* < 0.05). However, the BV/TV% showed a near-normal level when vitamin D was supplemented (99% of the control) (*p* > 0.05) (Figure 7C). Trabecular structural analyses showed that the reduction in bone volume was related to the reduction in trabecular thickness (90% of control when no vitamin D was supplemented) (*p* < 0.05), which appeared to be partially corrected when vitamin D was supplemented (94% of control) (*p* > 0.05) (Figure 7D). 

Furthermore, serum biochemical analyses showed that, whilst vitamin D supplementation had no effect in modifying MTX treatment-induced reduction in serum levels of bone formation marker ALP on both days 6 and 9 (*p* > 0.05 vs. MTX alone group) (Figure 7E), it displayed a strong tendency of attenuating MTX treatment-induced increase in serum levels of bone resorption marker CTX-1 on day 6 (*p* = 0.08 vs. MTX alone group) (Figure 7F), although this tendency disappeared on day 9. These data suggest that vitamin D supplementation (calcitriol given subcutaneously) can inhibit MTX chemotherapy-induced bone loss, which is likely due to its effect in suppressing bone resorption early after MTX treatment. 

## 4. Discussion

Childhood chemotherapy is known to significantly and negatively impact the bone health of childhood cancer patients and survivors, impairing bone growth, reducing bone mass, decreasing bone density, and increasing risks of fractures. With the known critical role of vitamin D in regulating Ca homeostasis and bone metabolism, there have been many previous studies that have focused on potential relationships between vitamin D levels and bone loss status in childhood cancer patients and survivors. Whilst these studies have demonstrated a high prevalence of lower serum levels of vitamin D in paediatric cancer patients and survivors [11,12,13,14,15,16,17,45], the underlying aetiology for the low vitamin D status has been unclear, and potential therapeutic effects of vitamin D supplementation in attenuating bone loss caused by chemotherapy have remained uncertain. Using a rat model of treatment with MTX (a most commonly used antimetabolite in childhood oncology), the current study demonstrated that MTX chemotherapy caused growth impairments (body weight loss, decreased body length gain and total tibial length), reduced serum vitamin D levels (to the extent of being deficient), altered bone turnover and reduced tibial trabecular bone volume in treated rats. MTX chemotherapy was found to cause significant intestinal mucosal damage with subsequent repair, altered vitamin D metabolism and altered expression of Ca-binding proteins in the intestine. Finally, subcutaneous vitamin D supplementation (with calcitriol) was found to significantly inhibit MTX chemotherapy-induced bone loss due to its effect in suppressing bone resorption.

### 4.1. Vitamin D Deficiency, Bone Turnover, Bone Loss, and Ca Homeostasis following MTX Chemotherapy

An adequate 25(OH)D level in the serum improves bone mineral density and helps to protect against osteoporosis and reduce risks of fractures [10]. Vitamin D deficiency contributes to the aetiology of osteomalacia (with the new bone or osteoid not being mineralised), causing rickets in the developing bones and osteoporosis in general [10,46]. Consistent with findings of lower vitamin D levels in childhood cancer patients and survivors [11,12,13,14,15,16,17,45] as well as in adult cancer patients following chemotherapy [47,48], the current study found significantly lower serum 25(OH)D levels in rats at all time points examined after MTX treatment (days 6, 9, and 14, being 42%, 58%, and 61% of the normal control level, respectively), to the extent of being deficient as they are lower than the widely accepted deficient level (<30 ng/mL) [37,38]. 

Although the serum vitamin D level of 25(OH)D < 30 ng/mL (with a prevalence of 75% in long-term survivors of ALL) was not found to be associated with lumbar spine-BMD outcomes in these ALL survivors [49], a lower serum 25(OH)D level (≤10 ng/mL) has been shown to be associated with lower BMD values in ALL survivors [50]. Similarly, a decreased BMD (at the lumbar spine, at the femoral neck and in the total hip) in adult survivors of stem cell transplantation and total body irradiation during childhood was found to be associated with a low level of serum 25(OH)D (14 ng/mL), suggesting that the lower BMD may at least in part be caused by 25(OH)D deficiency [51]. The current study showed that the proximal tibia of rats had lower trabecular bone mass after MTX treatment with a tendency of reduction in bone volume fraction on day 6, a significant decrease on day 9, and partial recovery on day 14, suggesting that the reduced vitamin D level after MTX chemotherapy was associated with the decreased bone mass. Consistently, the current study also observed significant changes in levels of serum markers of bone turnover. While levels of the bone formation marker ALP showed a tendency of reduction on day 6, they were significantly lower compared to the normal level on day 9. Levels of the bone resorption marker CTX-1 were significantly higher compared to the normal level on day 6 and showed a tendency to increase on day 9. The above findings on significant changes in levels of serum markers of bone turnover as well as bone volume are consistent with the significant changes in bone after MTX chemotherapy (reduced bone formation, increased resorption, and reduced trabecular bone volume) as observed previously [23,52]. Thus, our findings suggest that MTX chemotherapy in rats can significantly reduce serum vitamin D levels, which is associated with significant changes in bone turnover and bone loss.

However, despite the significant reductions in serum 25(OH)D levels following MTX treatment, serum calcium levels were found to be maintained in the current study, which perhaps could be explained by MTX chemotherapy-induced increased bone resorption (with an increased level of resorption marker CTX-1, as shown in the current study, and with the increased presence of bone-resorbing osteoclasts on bone surfaces, as shown previously [52]), resulting in release of bone tissue-derived Ca into the blood for maintaining Ca homeostasis. Our results suggest that following MTX chemotherapy, maintenance of serum calcium levels (for supporting functions of other vital tissues/organs) is achieved at least partially via the expense of increased bone turnover/bone resorption and bone loss.

Our data showed that by day 14, after the first of the five daily doses of MTX, rats rebounded well in many measurements, including the bone volume fraction (except for the serum vitamin D level and body/tibia lengths). Compared to survivors of childhood cancer, patients who can have long-term bone growth impairments and bone loss after receiving long-term intensive chemotherapy (usually with MTX in combination with other drugs, including glucocorticoids), this transient effect of MTX chemotherapy on bone volume fraction in rats could be due to the facts that the rats were treated only for a short-term, treated only with MTX, and/or were healthy (without bearing cancer). Further studies (e.g., with longer treatment terms or longer observation periods, with multiple-drug chemotherapy, and/or with cancer-bearing) are needed to investigate the treatment outcomes on serum vitamin D levels, whether the resultant bone defects can still be transient or irreversible, and whether they can persist into adulthood of rats. 

### 4.2. Vitamin D Deficiency, Bone Loss, and Altered Intestinal Vitamin D Metabolism Are Linked with MTX-Induced Intestinal Mucositis

While lower serum 25(OH)D levels have been found prevalent in cancer patients and survivors after chemotherapy, as discussed above, how chemotherapy negatively impacts 25(OH)D levels remains unclear, although some factors could potentially contribute to the reduced vitamin D levels, including dietary changes in cancer patients, reduced nutrient (including dietary vitamin D) absorption, and avoidance of sunlight [47]. Intestinal health supporting adequate absorption of nutrients, particularly dietary Ca and vitamin D, is critical for bone health [53], and central to the maintenance of Ca homeostasis by vitamin D is to increase the intestinal absorption of ingested Ca [22,46,54,55,56]. Vitamin D is critical in promoting the synthesis of Ca-carrying proteins in the small intestine, including TRPV6 and CALBINDIND9K [39,40]. During vitamin D deficiency, intestinal Ca absorption decreases [57]. The current study found that on day 6 after MTX chemotherapy, there was a significant increase in TRPV6 gene expression (as well as a trend of an increase in CALBINDIND9K expression) in the small intestine, suggesting presence of increased demand for Ca absorption locally in the intestine early after chemotherapy. This situation is analogous to increased expression levels of genes (TRPV5 and calbindinD-28K) responsible for Ca reabsorption in the proximal tubules of the kidney observed in mice with experimental chronic kidney disease [58]. Interestingly, the current study also found that the increased expression of TRPV6 and CALBINDIND9K on day 6 returned to normal levels on days 9 and 14, suggesting normalisation (during the later stage) of the intestinal calcium absorption demand (increased in the early stage). 

Since vitamin D metabolism has been found to be present locally in the intestine [41,42], and 1,25(OH)2D can be produced and serve a paracrine/autocrine function in the intestine [46,59,60], the current study has examined the effect of MTX chemotherapy on the expression in the intestine of two key metabolism enzymes, CYP27B1 and CYP24 or CYP24A1. CYP27B1, a 25D1α-hydroxylase, is responsible for converting the inactive and the major circulating form 25(OH)D to the active hormonal form 1,25(OH)2D (also called calcitriol), and CYP24 is the major vitamin D 24-hydroxylase that catabolises 25(OH)D and 1,25(OH)2D [41,42,43]. Suggesting increases in the activation of 25(OH)D to 1,25(OH)2D in the intestine, on day 6 after MTX chemotherapy, there was a significant increase in mRNA expression of CYP27B1 in the small intestine. Under the condition of reduced calcium absorption (as would be the case during mucosal damage on day 6 after MTX treatment), 25(OH)D is required to be activated by the activating enzyme CYP27B1 to 1,25(OH)2D to enhance the expression of calcium-binding proteins, particularly TRPV6 (known as one of the genes most responsive to 1,25(OH)2D) to increase calcium absorption. This situation is analogous to the higher CYP27B1 expression and local intestinal 1,25(OH)2D production observed during puberty, which may be a metabolic adaptation enhancing dietary Ca absorption at this time of human development [61].

On the other hand, interestingly, levels of expression of the vitamin D catabolising enzyme CYP24 were found to have no changes on day 6 but increased significantly on days 9 and 14, suggesting the presence of a local negative feedback mechanism: enhanced vitamin D catabolism in intestinal tissue during the healing stage on later time points (days 9 and 14) attempting to correct the higher local vitamin D activation status as shown present at the earlier time point day 6. This negative feedback mechanism with enhanced vitamin D catabolism is also consistent with the return of the increased levels of expression of calcium-carrying proteins present on day 6 to normal levels at later time points, on days 9 and 14, as described above. However, these changes in the expression of vitamin D metabolism enzymes, as well as the potential feedback mechanism present locally in the intestine during the intestinal healing stage, cannot reflect the persistently lower serum 25(OH)D levels on days 6, 9, and 14 after MTX chemotherapy. This, perhaps, can be explained by the facts that, although vitamin D metabolism can occur locally in the intestine as described above, 25(OH)D is produced mostly in the liver (by the hydroxylation enzyme CYP2R1), which is then converted to 1,25(OH)2D mainly in the kidney by the second hydroxylation enzyme CYP27B1 [22]. Further studies are required to investigate whether and how MTX chemotherapy would change vitamin D metabolism in these two major sites.

The current study also observed significant alterations in mucosal structure, goblet cell density, and activity of hydrolase sucrase in the small intestine after MTX treatment, suggesting that the significant changes in local intestine vitamin D metabolism were associated with MTX chemotherapy-induced intestinal mucosal damage and subsequent repair. Significant mucosal damage was observed in rats after the five once-daily MTX doses at 0.75 mg/kg (reduced villous height on days 6 and 9 and reduced crypt depth on day 6). In addition, consistent with the mucosal structural damages on days 6 and 9 and recovery on day 14, the current study also observed a significant loss of goblet cells in the villa on days 6 and 9 but a significant recovery on day 14. Goblet cells secrete mucin and glycoproteins for mucosal protection and, thus, can represent intestinal function and functional recovery [62]. The observations from this MTX treatment model are similar to the outcomes shown previously in rats receiving three daily high MTX doses (at 2.5 mg/kg) [25,34,63] or in mice after receiving a single high-MTX dose (300 mg/kg) [35]. Furthermore, we have demonstrated that MTX chemotherapy can significantly reduce the activity of intestinal mucosal hydrolase sucrase during the early stage (day 6) after MTX treatment, which can return to normal levels during the healing stage (days 9 and 14). These structural and functional changes observed in this and in previous studies in rodents are consistent with clinical observations of intestinal mucosal injury, malabsorption of nutrients, and diarrhoea in patients following MTX chemotherapy [64,65]. Our findings on MTX-induced intestinal damage and functional alterations are also consistent with our observations of body weight loss, decreased body length gain, and total tibial length in treated rats, suggesting that chemotherapy-induced intestinal mucositis and nutrient malabsorption are responsible at least in part for the defects in body growth and bone lengthening. 

Since MTX chemotherapy causes significant intestinal damage, which can lead to nutrient absorption defects, including absorption of dietary vitamin D and Ca, the vitamin D deficient status and bone loss seen in rats after MTX treatment should be contributed at least partially by defects in absorption of dietary vitamin D and Ca due to intestinal mucosal damage early after MTX treatment. In addition, significant changes in vitamin D metabolism observed in the intestine after MTX chemotherapy should also be driven by the significant intestinal mucosal damage and nutrient absorption defect (including Ca absorption). Specifically, the increased expression of vitamin D activation enzyme CYP27B1 and increased expression of calcium-binding proteins, particularly TRPV6, in the intestine are associated with mucosal damage and defects in absorbing dietary vitamin D and Ca during the early stage after MTX chemotherapy. Conversely, the increased expression of the vitamin D catabolism enzyme CYP24 and the recovery to normal levels in the expression of calcium-binding protein TRPV6 occurred at the mucosal healing stage (on later time points days 9 and 14) when intestinal absorption of dietary vitamin D and Ca return to normal levels. However, further studies are required to confirm the association of reduced serum vitamin D levels, altered intestinal vitamin D metabolism, and bone loss with MTX-induced intestinal damage. 

### 4.3. Potential Protective Effect of Vitamin D Supplementation and Action Mechanism

With our observations of significantly reduced serum vitamin D levels and bone loss following MTX chemotherapy, we subsequently investigated if vitamin D supplementation could attenuate MTX-induced bone loss in our rat model. We found that subcutaneous supplementation of calcitriol (the active form of vitamin D) was able to fully restore the serum vitamin D levels and attenuate proximal tibial local trabecular bone loss on day 9 after MTX treatment. In addition, while additional studies (e.g., gene expression analyses on the effects of vitamin D supplementation on bone formation and bone resorption) should be carried out to confirm our finding, our biochemical analyses of bone turnover markers suggest that the bone-rescuing effect of vitamin D supplementation was due to its action in suppressing bone resorption (as shown by a lower serum level of bone resorption marker CTX-1) early after MTX treatment. Similar to our findings, a recent study showed that vitamin D supplementation in vitamin D-deficient children and adolescents resulted in a more marked decrease in serum levels of bone resorption marker CTX-1 than the bone formation marker P1NP [66], and vitamin D supplementation was associated with a reduction in bone turnover markers in younger postmenopausal women with vitamin D deficiency [67]. 

However, the bone-rescuing effect of vitamin D supplementation was obtained in the current study when calcitriol was given subcutaneously. As the hormonally active form of vitamin D, calcitriol, can strongly stimulate intestinal calcium absorption and decrease bone turnover, it is the drug of choice in the treatment of calcitriol deficient conditions, including hypocalcemia in children, and it is also used to treat postmenopausal osteoporosis and the prevention of corticosteroid-induced osteoporosis [68,69]. In paediatric patients, calcitriol can be given either orally or via injections. Further studies are needed to investigate whether the positive effect of vitamin D supplementation observed in the current study can still be obtained if it is given orally during MTX chemotherapy since MTX chemotherapy is known to cause mucositis, being able to impair nutrient absorption (including dietary vitamin D). In addition, further animal studies are also required to compare potential therapeutic effects between calcitriol and the precursor vitamin D and to investigate the impact of their administration routes (orally vs. systemically) on chemotherapy-induced bone defects. 

While our study suggests that vitamin D supplementation may help to reduce or prevent bone loss in children receiving MTX chemotherapy, further studies (e.g., with longer terms of MTX treatment in animal models or with clinical studies) are needed to confirm our findings and to investigate effects of vitamin D supplementation on MTX chemotherapy-induced bone lengthening impairments. Furthermore, future studies are needed to investigate how MTX chemotherapy can change vitamin D receptor expression in the intestine, which would strengthen our mechanistic findings on the modulation of vitamin D action locally in the intestine with the upregulation (early after MTX treatment) of Ca-carrier proteins TRPV6 and CALBINDIND9k, which are vitamin D target genes [22,70]. Future studies are also needed to investigate whether vitamin D treatment can prevent or treat chemotherapy-induced intestinal damage (apart from the bone loss observed here). Since vitamin D can influence intestinal mucosal barrier function and regulate mucosal immunity [71,72,73] as well as suppress systemic inflammation [74], it is likely that vitamin D supplementation may prevent chemotherapy-induced intestinal mucositis or help the subsequent mucosal healing. In addition, since vitamin D can regulate the gut microbiome [71], and chemotherapy-induced intestinal microbiota dysbiosis has been shown to adversely impact intestinal mucosa homeostasis and aggravate intestinal mucositis [75], it is likely that vitamin D supplementation may attenuate chemotherapy-induced intestinal mucositis as it has been shown to be able to protect mice from dextran sodium sulfate-induced colitis due to its effect in modifying the gut microbial community [76]. 

## 5. Conclusions

**Figure 8:**
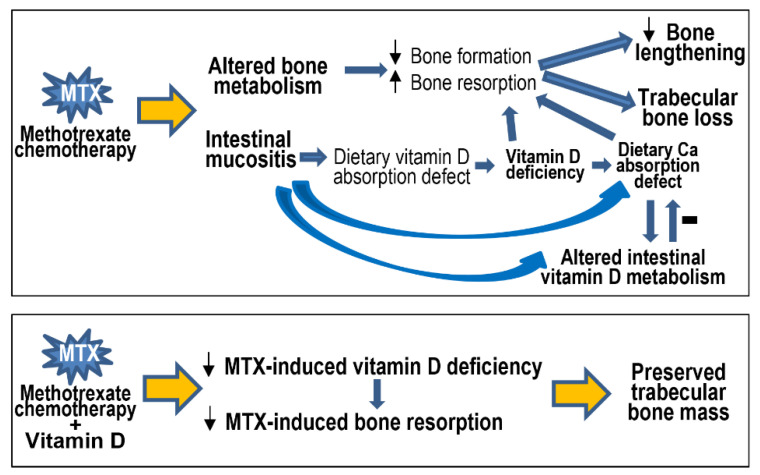
Schematic representation of methotrexate (MTX) chemotherapy-induced altered bone metabolism (decreased bone formation but increased bone resorption), bone growth impairments, and trabecular bone loss, which can also result indirectly and at least partially from MTX-induced intestinal mucosal damage (mucositis). Intestinal mucositis causes defects in absorption of dietary vitamin D (resulting in vitamin D deficiency) and calcium (Ca), and it alters intestinal vitamin D metabolism that can improve Ca absorption. Vitamin D supplementation can attenuate MTX-induced vitamin D deficiency and prevent the resultant bone resorption and bone loss. Small up and down arrows indicate increases and decreases, respectively.

The current study has demonstrated in a young rat model that MTX chemotherapy causes bone growth impairments, bone loss, vitamin D deficiency, and altered vitamin D metabolism in the intestine, which are associated with intestinal mucositis. Vitamin D supplementation subcutaneously (with calcitriol) can inhibit the resultant bone loss due to its effect in suppressing MTX-induced increased bone resorption (Figure 8). This study has provided mechanistic insight into vitamin D deficiency caused by MTX chemotherapy (which is commonly used in childhood oncology), and it has produced experimental evidence for the effectiveness of subcutaneous calcitriol supplementation in preventing chemotherapy-induced bone loss (commonly found in cancer patients and survivors).

## Data Availability

The data that support the findings of this study are available on reasonable request from the corresponding author.
