# Correction: Su et al. Methotrexate Chemotherapy Causes Growth Impairments, Vitamin D Deficiency, Bone Loss, and Altered Intestinal Metabolism—Effects of Calcitriol Supplementation. *Cancers* 2023, *15*, 4367

**DOI:** 10.3390/cancers17071207

**Published:** 2025-04-02

**Authors:** Yu-Wen Su, Alice M. C. Lee, Xukang Xu, Belinda Hua, Heather Tapp, Xue-Sen Wen, Cory J. Xian

**Affiliations:** 1 UniSA Clinical and Health Sciences, University of South Australia, Adelaide, SA 5001, Australia; yu-wen.su@unisa.edu.au (Y.-W.S.); alice.lee@unisa.edu.au (A.M.C.L.); xukang.xu@sa.gov.au (X.X.); belinda.hua@sa.gov.au (B.H.); 2 Department of Haematology & Oncology, Women’s and Children’s Hospital, North Adelaide, SA 5006, Australia; heather.tapp@sa.gov.au; 3 School of Pharmaceutical Sciences, Cheeloo College of Medicine, Shandong University, Jinan 250012, China; wsx@sdu.edu.cn

## Error in Figure and Text Correction

In the original article, there was a mistake in Figure 1B and Section 2.1.2 as published [1]. The vitamin (calcitriol) dosage should be 0.4 μg/kg rather than the incorrect 0.4 mg/kg. The corrected Figure 1B appears below. In Section 2.1.2, the text “0.4 mg/kg” should be corrected to “0.4 μg/kg”. The authors apologize for any inconvenience caused. The authors state that the scientific conclusions are unaffected. This correction was approved by the Academic Editor. The original publication has also been updated.

## Figures and Tables

**Figure 1:**
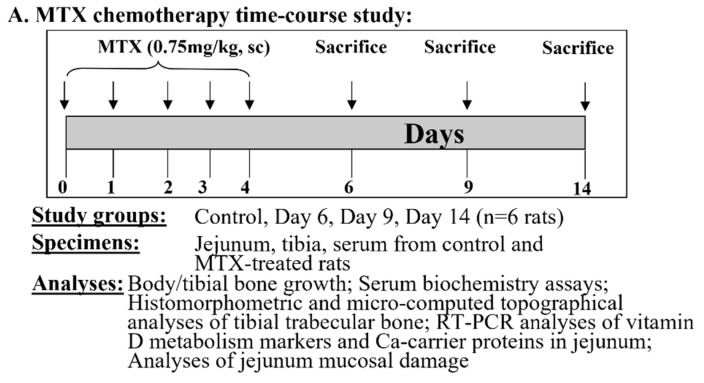

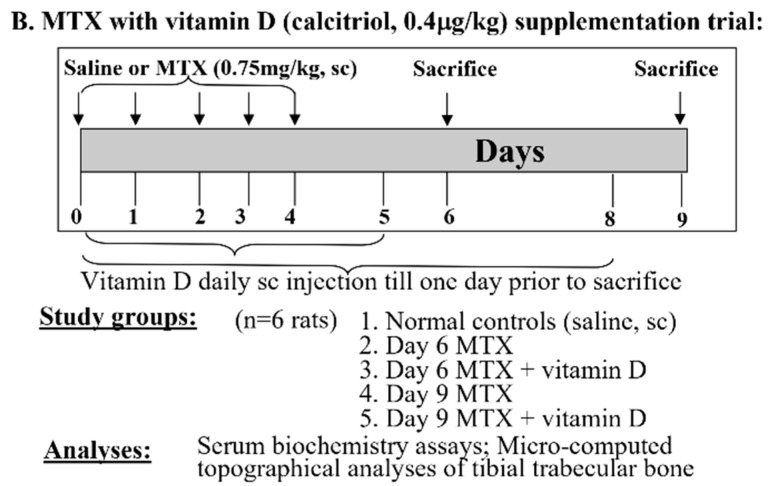
Design of this study. (**A**) Methotrexate (MTX) chemotherapy time-course study and (**B**) vitamin D (calcitriol) supplementation trial. Both MTX and vitamin D were given daily subcutaneously (sc) at doses specified.
